# Detection, transport, and retention of *Toxoplasma gondii* oocysts in saturated sandy porous media: Influence of electrolytes and natural organic matter in flow-through systems

**DOI:** 10.1371/journal.pone.0331812

**Published:** 2025-10-07

**Authors:** Christian P. Pullano, Mahsa Ghorbani, Timothy J. Mutty, Sohib Gouasmia, Coralie L’Ollivier, Jitender P. Dubey, Aurélien Dumètre, Christophe J. G. Darnault

**Affiliations:** 1 School of Civil and Environmental Engineering and Earth Sciences, Clemson University, Clemson, South Carolina, United States of America; 2 Aix Marseille University, IRD, AP-HM, SSA, RITMES, Marseille, France; 3 IHU-Méditerranée Infection, Marseille, France; 4 AP-HM, Parasitology Laboratory, Timone Hospital, Marseille, France; 5 United States Department of Agriculture, Agricultural Research Service, Animal Parasitic Diseases Laboratory, Beltsville Agricultural Research Center, Beltsville, Maryland, United States of America; 6 Aix Marseille University, CNRS, INSERM, LAI, Marseille, France; Bowen University, NIGERIA

## Abstract

Understanding the transport and retention of *Toxoplasma gondii* oocysts through soils and into ground and surface water is essential for determining the risk this parasite poses to water resources and human health worldwide. We studied here how various naturally occurring groundwater solutions containing different types of organic compounds (fulvic and humic acids) and electrolytes (NaCl, MgCl_2_, CaCl_2_) at different concentrations can affect the transport and retention of oocysts in engineered-saturated silica sand columns subjected to continuous flow to simulate the movement of groundwater through an aquifer. Breakthrough curve results from the qPCR analysis were then compared to non-reactive tracer tests to determine parameters that govern the transport of oocysts in saturated porous media. Though breakthrough of oocysts was observed in all tested solutions, higher ionic strength and ion valency resulted in greater oocyst retention. When both organic matter and electrolyte solutions were added to the systems, the electrolyte solutions displayed a far greater influence on parasite retention when compared to the influence of the organic matter alone. Collectively, this study demonstrates the pivotal role of soil groundwater solution chemistry in both the transport and retention of this important zoonotic parasite.

## Introduction

The protozoan *Toxoplasma gondii*, the causative agent of toxoplasmosis, is one of the most widespread zoonotic parasites, infecting between 30 and 80% of the population worldwide [1]. Its complex life cycle includes a sexual phase of multiplication in feline intestines (definitive hosts), which leads to the fecal excretion of oocysts, and an asexual phase in both definitive and intermediate hosts (birds and other mammals including humans), which results in the development and persistence of cysts preferentially in the brain, eyes, and muscles [2]. Humans can become infected by ingesting sporulated oocysts with vegetables and fruit grown in contact with contaminated soil and water or by drinking contaminated water, sometimes resulting in large-scale outbreaks [3]. They can also be infected by eating undercooked meat from meat-producing domestic animals and wild game containing tissue cysts of the parasite. Depending on the parasite genetic background and host immune status [4], *T. gondii* infections in humans may range from asymptomatic to severe ocular or cerebral diseases [2]. Immunocompromised individuals, such as AIDS patients or transplant recipients, and congenitally infected babies are at greater risk of severe diseases such as chorioretinitis [5] and encephalitis [6].

Physicochemical properties of oocysts and soils (texture, composition, water saturation and chemistry) in conjunction with the chemical composition of the groundwater matrix are key factors in the initial dynamics of oocyst spreading in soils and towards receiving groundwater and surface waters [3]. *Toxoplasma gondii* oocysts measure ~11 x 13 µm and have a specific gravity of 1.05-1.10 [7–9]. The oocyst wall is bilayered and contains ~90% proteins, mainly cross-linked cysteine- and tyrosine-rich proteins [9]. Because of its structure and molecular arrangement, the oocyst wall is very robust, with a Young’s modulus between 1–10 MPa, and is hard to rupture even after exposure to chemicals such as chlorinated disinfectants [10]. The wall has a zeta potential of −43.74 mV and an electrophoretic mobility in ultrapure water of −3.42 µm/s/V/cm, which likely favors repulsion between oocysts and surrounding particles [11].

The Derjaguin, Landau, Verwey, and Overbeek (DLVO) theory can be used to describe the attachment of biological colloids such as protozoan oocysts through saturated porous media [8,12–14]. In this theory, colloid immobilization is dictated by the double-layered interaction between colloid and grains, via London-van der Waals forces, and short-range repulsive forces. These forces are greatly affected by the ionic strength and pH of soil water, and the change of surface charge due to certain ions and organic matter compounds. In addition to the DLVO theory, the classic colloid filtration model (CFM) can also describe the process by which colloids are adsorbed onto the grain surface and strained by the grains due to the fluid motion within the system and the charge of the colloid around the surface of grains [15].

Variations in solution chemistry can significantly influence the forces that govern the transport and retention of oocysts from other protozoan parasites such as *Cryptosporidium parvum* in environmental and subsurface systems. Previous research has extensively investigated the transport and retention of oocysts in diverse porous media, including sandy soils, aquifer sediments, repacked soil columns, and variably saturated subsurface environments. These studies have demonstrated how factors such as soil texture, surface chemistry, preferential flow pathways, and solution composition affect oocyst mobility and retention [11,13,16–21]. Key mechanisms include electrostatic repulsion, van der Waals attraction, steric hindrance, and divalent cation bridging, all of which affect how oocysts deposit onto soil particle surfaces [20,22–24]. Natural organic matter (NOM) has also been shown to modify surface charge interactions and influence oocyst attachment and transport behavior [11,25]. Steric interactions can reduce the impact of ionic strength and electrolyte composition on oocyst surfaces, effectively minimizing the Debye length [23,26]. Experimental studies have demonstrated that calcium ions (Ca²⁺) promote greater deposition of *C. parvum* oocysts onto silica surfaces compared to sodium ions (Na⁺) [23,27]. Electrostatic interactions are generally considered more influential than hydrophobic effects in controlling oocyst transport through porous media [28–31].

Straining, attachment, and detachment processes are also recognized as critical factors limiting oocyst mobility in soils and subsurface environments [16,32]. These insights have informed the development of transport models that account for both fast and slow kinetic attachment processes [30]. Additionally, continuous-time random walk models have been used to describe the influence of collector surface heterogeneity and the stochastic nature of oocyst movement through porous media over space and time [33].

Using homogeneously packed-soil columns spiked with *T. gondii* oocysts and qPCR analyses, we previously demonstrated that straining and adsorption of oocysts appear central in controlling the transport of the parasites throughout natural soils subjected to artificial rainfall and surfactant application [13]. However, this configuration did not allow us to investigate the role of water chemistry and dissolved natural organic matter in the transport of oocysts in saturated porous media. Here, we tested the hypothesis that the transport of oocysts in saturated porous media was (i) enhanced in the presence of organic compounds due to lower adsorption rates and (ii) diminished due to higher adsorption rates because of the increased valence and ionic strength of electrolyte solutions. To this end, engineered silica-sand columns were spiked with oocysts and subjected to continuous flow of solutions containing different types and concentrations of organic compounds (fulvic and humic acids) and electrolyte solutions (NaCl, MgCl_2_, CaCl_2_). Oocyst breakthrough was quantified in columns effluents by qPCR and compared to non-reactive tracer and KCl control conditions to determine parameters that dictate the parasite transport in such saturated porous media.

## Materials and methods

### *Toxoplasma gondii* oocysts

*Toxoplasma gondii* oocysts of the ME 49 strain (genotype II) were obtained from feces of a 3-month old male cat at the United States Department of Agriculture Agricultural Research Service (USDA-ARS) Beltsville Agricultural Research Center (BARC), Beltsville, Maryland, United States, as described [34]. The cat was born and raised at BARC facility in a SPF (Specific Pathogen Free) colony. This experiment was performed in 2017, before the redirection of *Toxoplasma* research at the USDA. All biosafety and ethical procedures used protocol 012 approved by the Beltsville Area Animal Care and Use Committee (BAACUC), United States Department of Agriculture, Beltsville, Maryland. The cat was asymptomatic at all times and was euthanized 8 days after feeding tissue cysts, using the euthanasia procedure approved by BAACUC (ketamine at 10 mg/kg body weight in combination with xylazine at 1 mg/kg body weight administered intramuscularly to anesthetize cats before intracardiac administration of a barbiturate overdose (Beuthanasia, Schering-Plough Animal Health) administered intracardiac at 1 mg/4.5 kg body weight). Sporulated oocysts were processed as previously described [13]. Oocysts were stored in 2% sulfuric acid until used.

### Groundwater chemistry

A 1 mM potassium chloride (KCl) solution (ACS Grade, CAS# 7447-40-7, Solon, OH) was used to simulate natural groundwater chemistry and served as the control solution; all other experimental solutions were prepared using this 1 mM KCl solution as their aqueous base.

The organic compounds used in this experiment include humic acid (IHSS Humic Acid Standard III 3S101H, St. Paul, MN) and fulvic acid (IHSS Fulvic Acid Standard II 2S101F, St. Paul, MN) collected from the Suwannee River. Concentrations of 1 and 10 mg/L were used, which are within the expected natural groundwater concentrations for dissolved organic compounds. The monovalent and divalent ionic salts solutions were prepared at 1- and 10-mM final concentrations using sodium chloride, magnesium chloride hexahydrate (ACS Grade, CAS#7791-18-6, Solon, OH), and calcium chloride anhydrous (ACS Grade, CAS#10043-52-4, Solon, OH). Solutions containing 1- or 10-mM calcium chloride and 1 mg/L humic acid were also tested.

Solutions were made in 2-liter batches to fill at least ten pore volumes (PV) of the experimental transport columns.. In addition, a 10 mM solution of potassium bromide (KBr) was used as a non-reactive conservative tracer to characterize flow behavior and hydrodynamic properties of the column. This test was conducted in columns used exclusively for tracer testing. The tracer was measured using an Orion™ Bromide Electrode (ThermoFisher Scientific, Waltham, Massachusetts, USA).

### Oocyst pulse

Oocysts from the stock suspension were washed three times in ultra deionized water by centrifugation at 2,500 g for 15 min at 10°C to remove sulfuric acid. After the final washing step, the supernatant was brought to 10 mL with water. The sample was then vortexed and pipetted into four separate tubes of 2.5 mL, one for each of the four columns. Each of the four 2.5 mL samples was then added to a 50-mL tube and the balance was filled with 47.5 mL of KCl background solution. The tubes were vortexed and allowed to equilibrate at 4°C for 24 hr prior to conducting the transport experiments. Five milliliters from each tube were collected to quantify the initial concentration within the pulse by qPCR (calculated at 9,262,058 oocysts/mL). A 45-mL KBr solution without oocysts was used as a non-reactive tracer test.

### Column experiments

Oocyst transport experiments were conducted using engineered-saturated silica sand columns subjected to continuous flow. For this, Spectra/Chroma® glass columns with dimensions 5 cm in diameter by 20 cm in length were packed with a 20 x 40 US silica sand with a particle size between 0.420 and 0.841 mm, an effective porosity of 35%, and a particle density of 2.65 g/cm^3^. Columns were placed upright with the inlet at the bottom and outlet at the top to reduce the effects of settling within the columns. SpectraMesh® 297-µm polypropylene filters were used to hold the silica sand in place and prevent clogging of the tubing connected to the columns. Column influent was fed with 1.6 mm internal diameter silicone Masterflex® tubing. Tubing was connected to a Masterflex® L/S Peristaltic Pump model 7523−60 set at a constant flow rate of 250 mL/hr equating to an average seepage velocity of 36.4 cm/hr. This velocity was chosen based on natural flow velocities observed in groundwater systems containing medium grained sand.

The experimental setup involved flooding the columns for an inundation period with 1 PV of groundwater background solution and allowing the columns to equilibrate to the solution chemistry for 2 hr. After the inundation period, another PV was flushed through the system to allow the groundwater chemistry within the system to equilibrate and remain constant after coating sand grains. After flushing those 2 PV through the system, the oocyst pulse was injected into the column using the peristaltic pump. Seven PV of groundwater solution then followed this pulse to flush the pulse through the system. Collection of the effluent exiting the system began at the beginning of pulse injection. Effluent exiting the system was collected in 1/10 of a PV increment of 15 mL for the first 5 PV and 1/3 of a PV of 50 mL for the last 2 PV. The same procedure was performed for all transport columns involving organic solutions, electrolyte solutions, KCl control and non-reactive KBr tracer solutions. Column experiments were conducted in duplicate, with newly prepared sand packed as fresh porous media for each replicate. A total of 28 columns was performed in this study. This generated 56 effluent samples per column totaling 1,568 total samples collected.

### DNA extraction and quantification of oocysts by qPCR in column effluents

Half of the total sample effluents collected from column experiments were submitted to a series of centrifugation steps to concentrate the oocysts down to a 50 µL volume for the subsequent DNA extraction. To weaken the oocyst walls and enhance DNA extraction, samples were subjected to six cycles of freezing at −80°C for 5 min and thawing at 90°C for 5 min prior to sonication for 10 min. DNA was extracted using a E.Z.N.A.® Tissue DNA kit (OMEGA Bio-Tek, #D3396-02, VWR International S.A.S., Strasbourg, France). The real-time PCR analysis targeted a 529 bp repeat regions (REP529, GenBank accession no. AF487550) of *T. gondii* [35]. Real-time PCR reactions were conducted using a Roche’s LightCycler 480 with a final volume of 25 μL containing 1X LightCycler^TM^ 480 Probes Master (ROCHE Diagnostics, France), 0.5 μM of each required primer [13], 0.25 μM of Taqman probe, 0.5µl of 1% bovine serum albumin (BSA), and 2 μL of template DNA. Thermal cycling conditions were 95°C for 10 min, 45 cycles of 10 sec at 95°C, 30 sec at 58°C and 10 sec at 72°C. qPCR results were expressed in cycle threshold values (Ct) and the oocyst number was calculated using a sample curve created by five 10-fold serial dilutions of a sample with a known oocyst concentration.

### Mathematical analysis

It is common to compare breakthrough curves (BTCs) of saturated flow column experiments using moment calculations instead of comparing the entire distribution of the BTCs [13]. Three moments are typically used for these calculations to describe the mean residence time, variance, and skewness of the results. To quantitatively analyze the results and make them more useful for the public, decision-makers, and stakeholders, the moments were calculated for each replicate of the BTCs. The first moment is the mean residence time, as defined by the equation:


$$t_{m}=\int_{\text{0}}^{\infty }tE(t)dt$$ (1)

where:

$t_{m}$: Mean residence time [time]

t: Time variable [time]

E(t): Residence time distribution (RTD) [1/time] or E curve function.

This equation describes the mean residence time $t_{m}$ for a particle, solute, or tracer within the column reactor.

The E curve function is described by the equation:


$$E(t)=\frac{C(t)}{\int_{\text{0}}^{\infty }C(t)dt}$$ (2)

where:

E(t): Residence time distribution (RTD) [1/time] or E curve function

C(t): Concentration of oocysts or tracer in the influent at time t [oocysts/mL]

t: Time variable [time]

The E curve equation describes the concentration C(t) of the oocysts and tracer with respect to time over the integral of the concentration of oocysts and tracer with respect to time.

The second moment, or variance, is taken about the mean residence time $t_{m}$, and is equal to the square of the standard deviation of the breakthrough curve. It is defined by the equation:


$$\sigma^{\text{2}}=\int_{\text{0}}^{\infty }{\left(t-t_{m}\right)}^{\text{2}}E(t)dt$$ (3)

where:

$\sigma^{\text{2}}$: Variance of the residence time distribution [time^2^]

t: Time variable [time]

$t_{m}$: Mean residence time [time]

E(t): Residence time distribution (RTD) [1/time] or E curve function

This second moment indicates the spread of the distribution of the breakthrough concentrations. Larger second moments indicate a greater distribution of the spread of results from the mean residence time of the colloids or tracer within the systems.

The third moment, also measured about the mean residence time, represents the skewness of the data. It is defined by the equation:


$$S^{\text{3}}=\frac{\text{1}}{\sigma^{\frac{\text{3}}{\text{2}}}}\int_{\text{0}}^{\infty }{\left(t-t_{m}\right)}^{\text{3}}E(t)dt$$ (4)

where:

$S^{\text{2}}$: Skewness [dimensionless]

t: Time variable [time]

$t_{m}$: Mean residence time [time]

E(t): Residence time distribution (RTD) [1/time] or E curve function

σ: Standard deviation (square root of variance) [time]

E(t): Residence time distribution (RTD) [1/time] or E curve function

The third moment measures the level at which a distribution is skewed in one direction or another with respect to the mean distribution of the BTCs. A positive value for the skewness, which results in a tailing effect of the BTC, is the expected result for saturated flow systems which react with the porous media.

## Results

### Baseline flow rates through the saturated porous media

**Fig 1:**
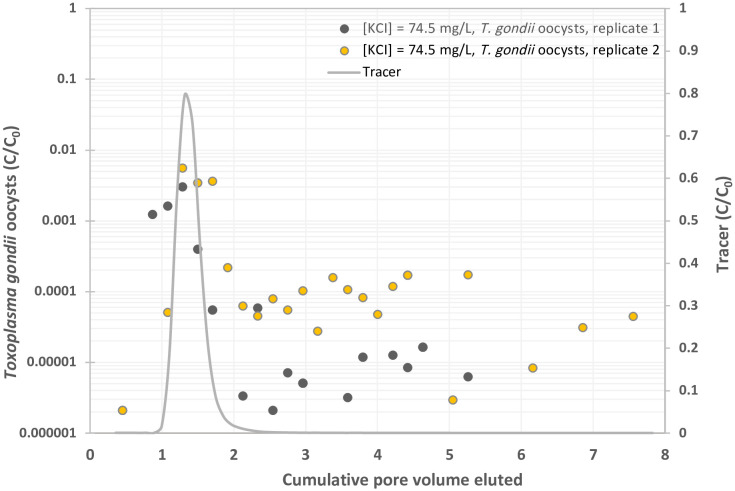
Experimental BTCs of *Toxoplasma gondii* oocysts in suspension in 1 mM KCl solution, y-axis represents the normalized effluent concentration $\frac{C}{C_{0}}$ and x-axis represents dimensionless time expressed as pore volumes, which are equivalent to the volume of fluid injected divided by the total pore volume of the column.

**Fig 2:**
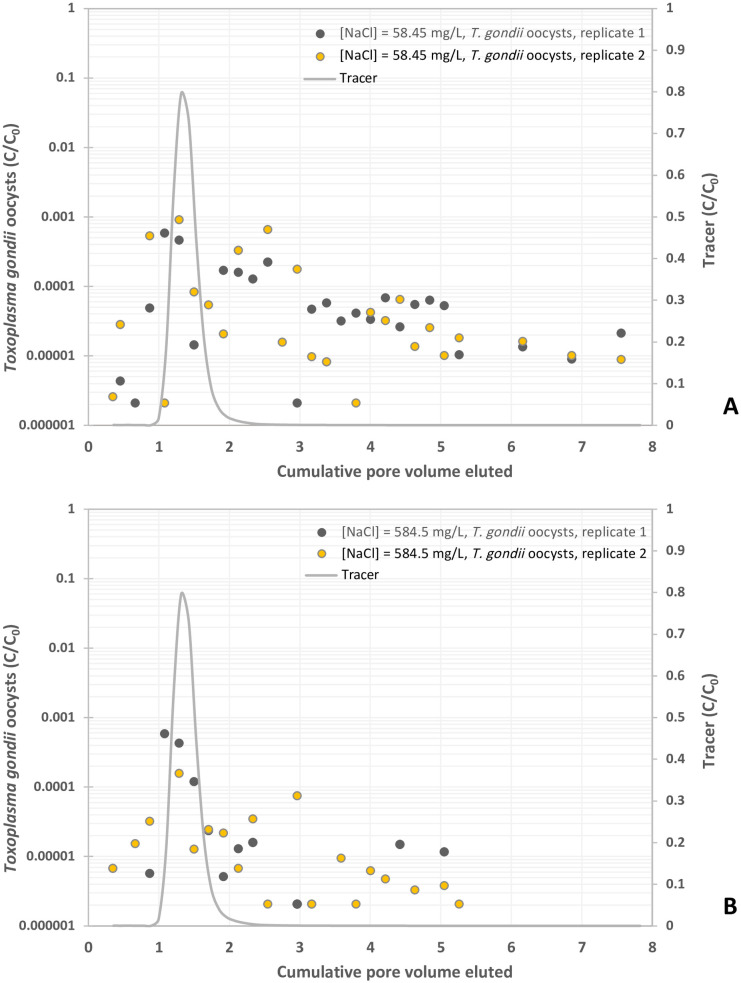
Experimental BTCs of *Toxoplasma gondii* oocysts in suspension of NaCl at different concentrations, y-axis represents the normalized effluent concentration $\frac{C}{C_{0}}$ and x-axis represents dimensionless time expressed as pore volumes, which are equivalent to the volume of fluid injected divided by the total pore volume of the column. (A) NaCl = 1 mM, and (B) NaCl = 10 mM.

**Fig 3:**
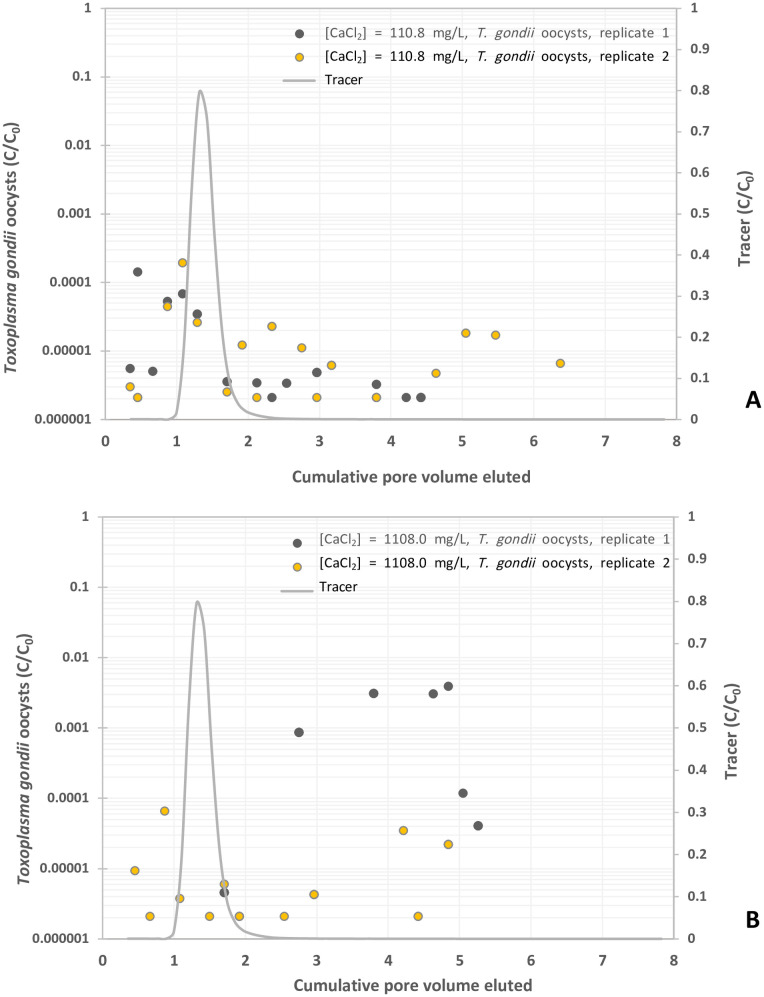
Experimental BTCs of *Toxoplasma gondii* oocysts in suspension of CaCl_2_ at different concentrations, y-axis represents the normalized effluent concentration $\frac{C}{C_{0}}$ and x-axis represents dimensionless time expressed as pore volumes, which are equivalent to the volume of fluid injected divided by the total pore volume of the column. (A) CaCl_2 _= 1 mM, and (B) CaCl_2 _= 10 mM.

**Fig 4:**
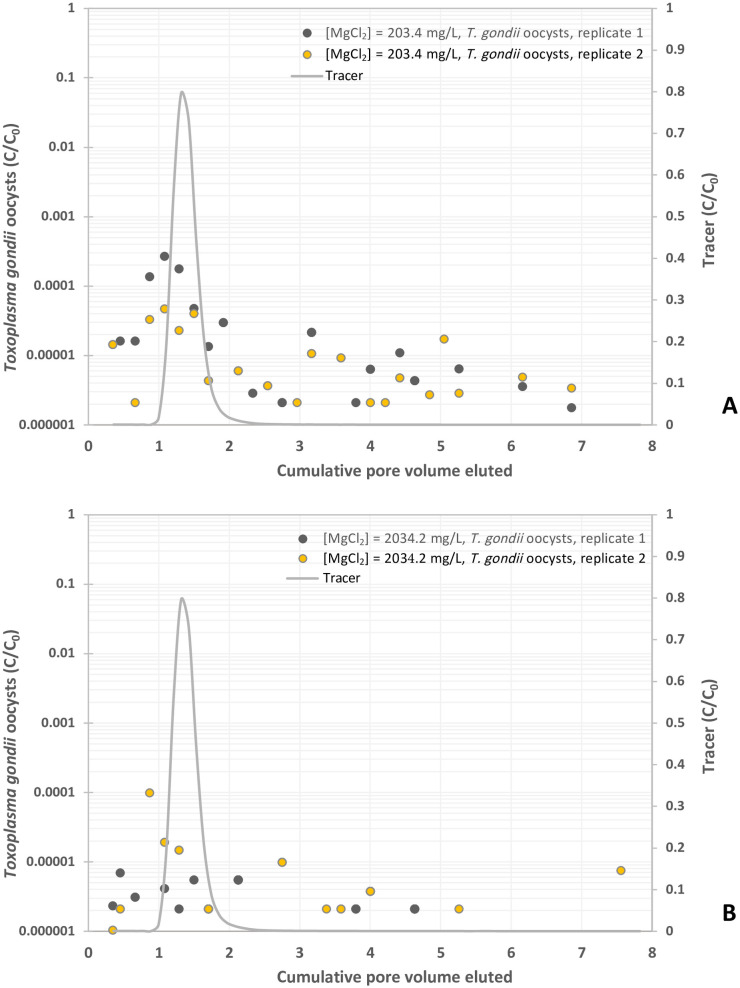
Experimental BTCs of *Toxoplasma gondii* oocysts in suspension of MgCl_2_ at different concentrations, y-axis represents the normalized effluent concentration $\frac{C}{C_{0}}$ and x-axis represents dimensionless time expressed as pore volumes, which are equivalent to the volume of fluid injected divided by the total pore volume of the column. (A) MgCl_2 _= 1 mM, and (B) MgCl_2 _= 10 mM.

**Fig 5:**
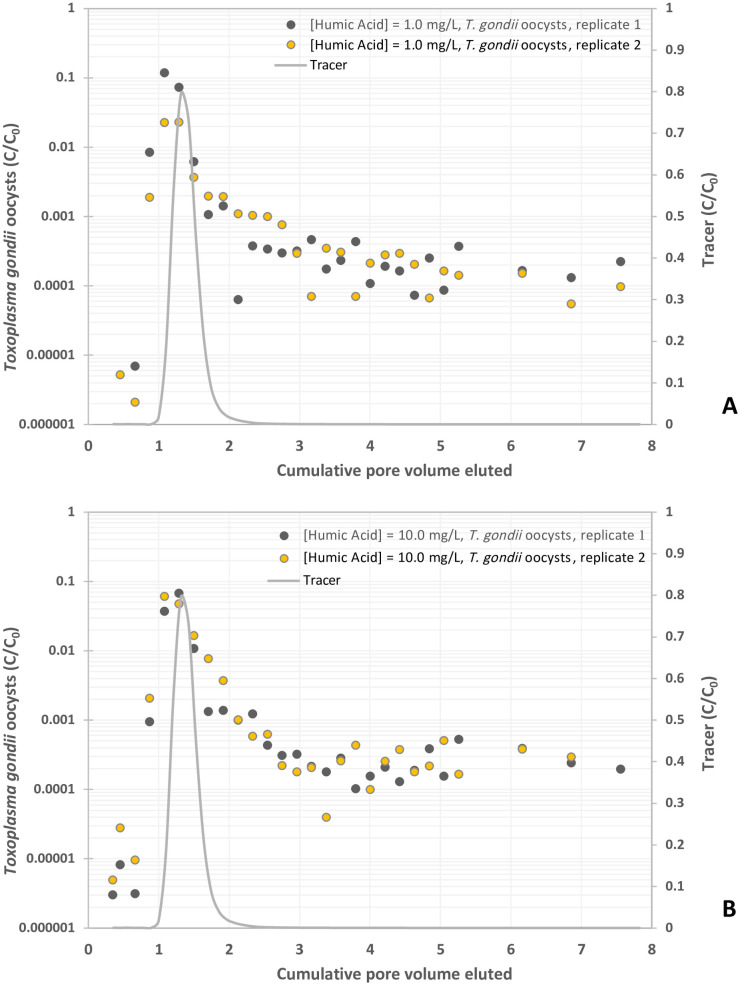
Experimental BTCs of *Toxoplasma gondii* oocysts in suspension of humic acid at different concentrations, y-axis represents the normalized effluent concentration $\frac{C}{C_{0}}$ and x-axis represents dimensionless time expressed as pore volumes, which are equivalent to the volume of fluid injected divided by the total pore volume of the column. (A) Humic acid = 1 mg/L, and (B) Humic acid = 10 mg/L.

**Fig 6:**
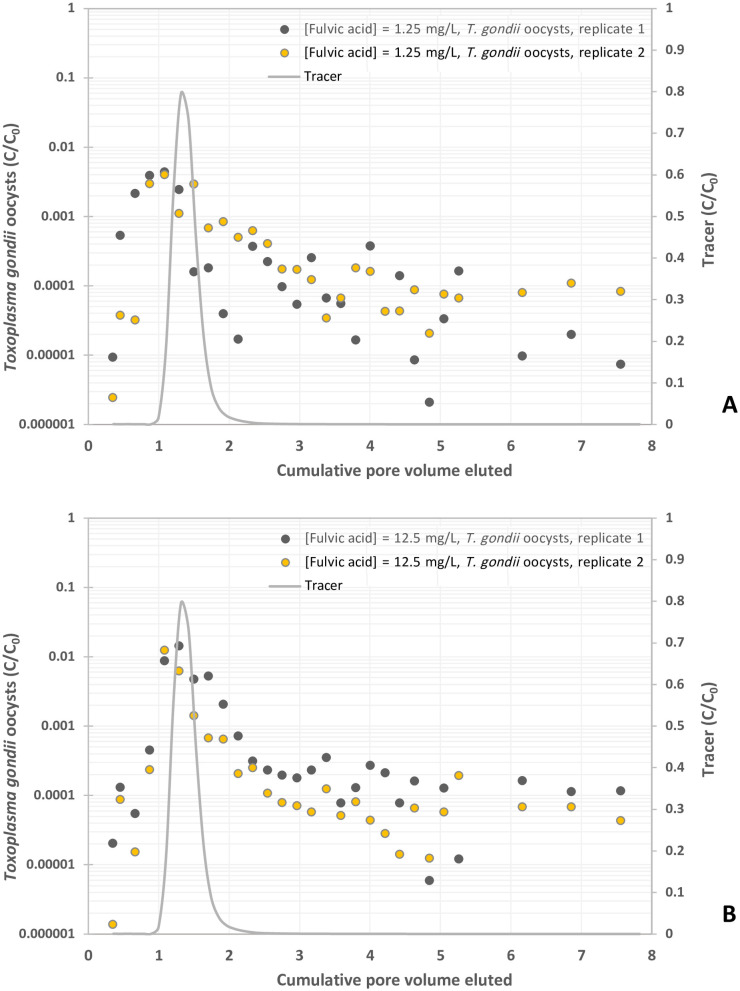
Experimental BTCs of *Toxoplasma gondii* oocysts in suspension of fulvic acid at different concentrations, y-axis represents the normalized effluent concentration $\frac{C}{C_{0}}$ and x-axis represents dimensionless time expressed as pore volumes, which are equivalent to the volume of fluid injected divided by the total pore volume of the column. (A) Fulvic acid = 1. mg/L, and (B) Fulvic acid = 10 mg/L.

**Fig 7:**
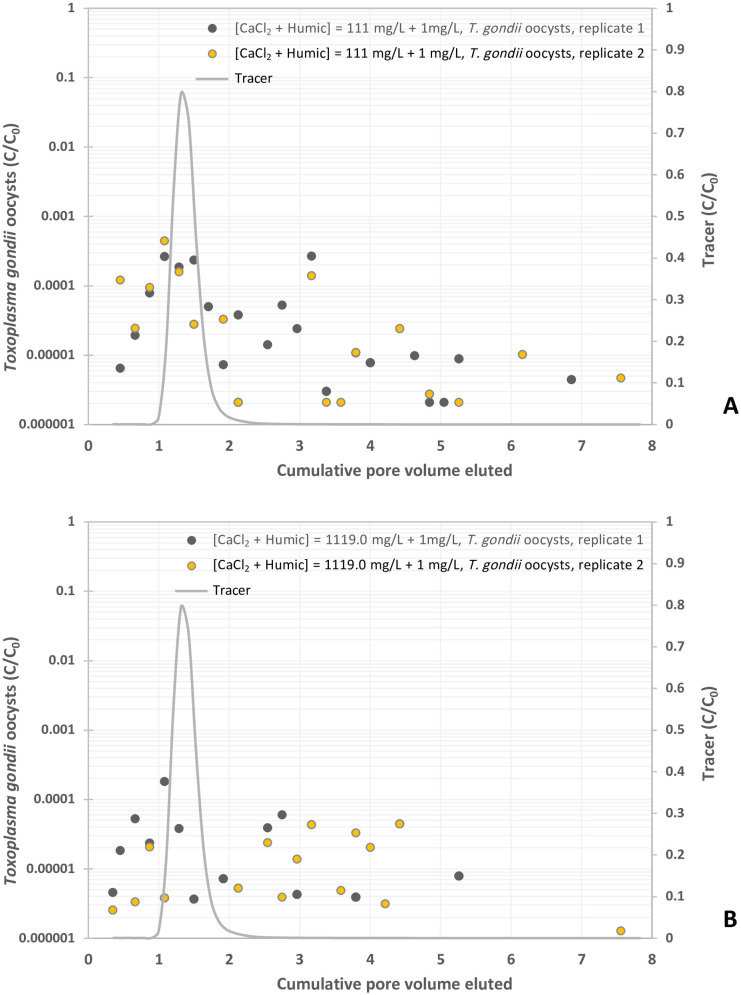
Experimental BTCs of *Toxoplasma gondii* oocysts in suspension of CaCl_2_ – humic acid at different concentrations, y-axis represents the normalized effluent concentration $\frac{C}{C_{0}}$ and x-axis represents dimensionless time expressed as pore volumes, which are equivalent to the volume of fluid injected divided by the total pore volume of the column. (A) CaCl_2_ – Humic acid = 1 mM CaCl_2_ and 1 mg/L Humic acid, and (B) CaCl_2_ – Humic acid = 10 mM CaCl_2_ and 1 mg/L Humic acid.

A nonreactive KBr column experiment BTC was used to establish baseline flow rates through the sandy saturated porous media for comparison of the oocyst column transport experiments. The nonreactive KBr tracer BTC yielded an average peak at 1.32 PV, with an average peak concentration C/C_0_ of 0.8 and an average total C/C_0_ breakthrough recovery of 95.3% where C_0_ is the concentration of oocysts in the input solution (Figs 1–7 and Table 1).

**Table 1:** Breakthrough curve key parameters related to *Toxoplasma gondii* oocysts and KBr tracer, and recovery data for *T. gondii* oocysts in leachates for each groundwater chemistry solutions Light grey, conditions for which oocysts are retained in systems compared to the KCl control; Dark grey, conditions for which oocysts were transported throughout the systems compared to the KCl control.

Systems	Replicate number	First detection of oocysts in column effluent [pore volumes]	Pore volume of the peak concentration of oocysts in column breakthrough [pore volumes]	Maximum peak concentration of oocysts in column breakthrough (oocysts/ml of effluent/ oocysts/ml in pulse) [C/C_0_]	Percentage mass balance comparison to KCl mass balance average (%)	Percentage mass balance of oocysts or tracer [total oocysts or tracer in effluent/ total oocysts or tracer in the pulse] (%)	Percentage mass balance of oocysts or tracer [total oocysts or tracer in effluent/ total oocysts or tracer in the pulse] average of the two replicates (%)	Percentage mass balance of oocysts compared to KCl control (%)
KBr 10 mM	1	0.36	1.32	0.73	–	92.825	–	–
	2	0.36	1.32	0.87	–	97.681	–	–
KCl 1 mM	1	0.87	1.29	3.01 x 10^−3^	61.85	0.431	0.697	100
	2	0.45	1.29	5.59 x 10^−3^	138.15	0.963		
NaCl 1 mM	1	0.35	1.08	5.86 x 10^−4^	23.55	0.164	0.190	27.26
	2	0.35	1.29	9.21 x 10^−4^	31.01	0.216		
NaCl 10 mM	1	0.87	1.08	5.95 x 10^−4^	11.94	0.083	0.064	9.18
	2	0.35	1.29	1.59 x 10^−4^	6.45	0.045		
CaCl_2_ 1 mM	1	0.35	0.45	1.42 x 10^−4^	2.59	0.018	0.023	3.30
	2	0.35	1.08	1.93 x 10^−4^	4.05	0.028		
CaCl_2_ 10 mM	1	1.71	4.84	3.93 x 10^−3^	1.06	0.007	0.008	1.15
	2	0.45	0.87	6.55 x 10^−5^	1.49	0.010		
MgCl_2_ 1 mM	1	0.45	1.08	2.71 x 10^−4^	7.49	0.052	0.035	5.02
	2	0.35	1.08	4.73 x 10^−5^	2.62	0.018		
MgCl_2_ 10 mM	1	0.35	0.45	7.02 x 10^−6^	0.33	0.002	0.007	1.00
	2	0.35	0.87	9.90 x 10^−5^	1.78	0.012		
Humic acid 1 mg/L	1	0.66	1.08	1.19 x 10^−1^	2067.40	14.404	9.294	1333, 43
	2	0.35	1.29	2.29 x 10^−2^	600.68	4.185		
Humic acid 10 mg/L	1	0.35	1.29	6.78 x 10^−2^	1236.10	8.612	9.249	1326.97
	2	0.35	1.08	6.12 x 10^−2^	1419.00	9.887		
Fulvic acid 1 mg/L	1	0.35	1.08	4.41 x 10^−3^	150.07	1.046	1.074	154.09
	2	0.35	1.08	4.04 x 10^−3^	158.13	1.102		
Fulvic acid 10 mg/L	1	0.35	1.29	1.45 x 10^−2^	392.76	2.736	2.172	311.62
	2	0.35	1.08	1.26 x 10^−2^	230.95	1.609		
CaCl_2_ 1 mM + Humic acid 1 mg/L	1	0.35	1.08	2.66 x 10^−4^	12.50	0.087	0.080	11.48
	2	0.35	1.08	4.46 x 10^−4^	10.47	0.073		
CaCl_2_ 10 mM + Humic acid 1 mg/L	1	0.35	1.08	1.83 x 10^−4^	4.24	0.030	0.023	3.30
	2	0.35	4.42	4.48 x 10^−5^	2.24	0.016		

### Oocyst transport in KCl control solution

Oocyst breakthrough in the 1 mM KCl control solution was observed prior to the peak of the nonreactive KBr tracer curve (Fig 1). Oocysts were detected in the first sampled volume from 0.45 to 0.87 PV and peaked at 1.29 PV, with concentrations ranging from 3.0 x 10^−3^ to 5.6 x 10^−3^ C/C_0_ (Table 1), then followed by a decrease around 1.5 x 10^−5^ C/C_0_ (Fig 1). On average, oocyst breakthrough in 1 mM KCl relative to the total pulse was 0.697% (Table 1).

### Effects of electrolytes on the transport of oocysts

Oocyst breakthrough in 1 mM and 10 mM NaCl solutions was observed prior to the peak of the KBr tracer curve (Fig 2A and 2B). For the 1 mM NaCl (Fig 2A), oocyst breakthrough was detected at 0.35 PV and peaked from 1.08 to 1.29 PV at concentrations ranging from 5.9 x 10^−4^ to 9.2 x 10^−4^ C/C_0_ prior decreasing to C/C_0_ values between 2.1 x 10^−6^ and 1 x 10^−4^ (Table 1). In comparison with 1 mM NaCl, the 10 mM NaCl solution yielded the same graph shape but resulted in a lower transport of oocysts (Fig 2B). Oocysts were first detected between 0.35 and 0.87 PV and peaked at 1.08 to 1.29 PV, with concentrations ranging from 1.6 x 10^−4^ to 5.9 x 10^−4^ C/C_0_ prior to decreasing at around 1.5 x 10^−4^ C/C_0_ (Fig 2B and Table 1). On average, oocyst breakthrough relative to the total pulse was 0.190% and 0.064% in 1 mM and 10 mM NaCl respectively (Table 1). Compared to the KCl control solution, the addition of 1 mM and 10 mM NaCl resulted in oocyst breakthroughs of 27.3% and 9.2% respectively (Table 1).

Oocyst breakthrough in 1 mM CaCl_2_ occurred prior to the peak of the KBr tracer curve (Fig 3A) and, in 10 mM CaCl_2_, after the tracer for one column experiment and before for the duplicate (Fig 3B). For the 1 mM CaCl_2_ solution, oocysts were detected first at 0.35 PV and peaked at 0.45 to 1.08 PV, with concentrations ranging from 1.4 x 10^−4^ to 1.9 x 10^−4^ C/C_0_ prior trailing off at 3.5 x 10^−6^ C/C_0_ (Fig 3A and Table 1). In comparison with 1 mM CaCl_2_ solution, the 10 mM CaCl_2_ solution yielded two graph shapes with higher oocyst concentrations and a delayed breakthrough (Fig 3B). Oocysts were first detected at 0.45 and 1.71 PV and peaked from 0.87 to 4.84 PV, with concentrations ranging from 6.6 x 10^−5^ to 3.9 x 10^−3^ C/C_0_ (Table 1). Oocyst breakthrough did not then appear to decrease to around 1.1 x 10^−4^ C/C_0_ up to 7.5 PV (Fig 3B). On average, oocyst breakthrough relative to the total pulse was 0.023% and 0.008% in 1 mM and 10 mM CaCl_2_ respectively (Table 1). Compared to the KCl control solution, the addition of 1 mM and 10 mM CaCl_2_ resulted in oocyst breakthroughs of 3.3% and 1.1% respectively (Table 1). Increasing the concentration of CaCl_2_ increased oocyst attachment and retention, reducing their breakthrough.

Oocyst breakthrough in both MgCl_2_ solutions (1 and 10 mM) occurred prior to the peak of the KBr tracer curve (Fig 4A and 4B). Oocysts in 1 mM MgCl_2_ solution were detected between 0.35 and 0.45 PV and peaked at 1.08 PV, with concentrations ranging from 4.7 x 10^−5^ to 2.7 x 10^−4^ C/C_0_ prior to decrease to 2.2 x 10^−5^ C/C_0_ (Fig 4A and Table 1). The 10 mM MgCl_2_ solution yielded the same graph shape with lower overall concentrations of oocysts in sampled effluents (Fig 4B). Oocysts were first detected at 0.35 PV and peaked at PV ranging from 0.45 to 0.87 with concentrations ranging from 7.0 x 10^−6^ to 9.9 x 10^−5^ C/C_0_ (Fig 4B and Table 1). The oocyst breakthrough then decreased around 9.9 x 10^−6^ C/C_0_ values (Fig 4B). The amount of oocysts compared to the total pulse was 0.035% and 0.007% in 1- and 10-mM solution respectively (Table 1). Compared to the KCl control solution, adding 1 mM and 10 mM MgCl_2_ resulted in oocyst breakthroughs of 5.0% and 1.0% respectively (Table 1). Increasing MgCl_2_ concentration therefore decreased oocyst breakthrough and increased their retention.

When comparing the mean residence time of oocysts from electrolyte experiments, all BTCs appear to be within the same range as the control KCl BTCs (Table 2). There was a delayed oocyst breakthrough for 1 mM NaCl and 10 mM CaCl_2_ and an enhanced breakthrough for 10 mM NaCl and 1 mM CaCl_2_ compared to KCl (1.1-1.4 PV). In 1 mM NaCl and 10 mM CaCl_2_, the mean residence time increased to 1.7-1.8 and 1.4-4.5 PV respectively, while it decreased to 1.1-1.2 PV and 0.8-1.0 PV for 10 mM NaCl and 1 mM CaCl_2_ respectively. Most of oocyst BTCs in electrolyte solutions had a greater variance than KCl, with the higher ionic strength solutions exhibiting the highest average variance (Table 2). The third moment of skewness of the BTCs for solutions containing electrolytes displayed greater positive values, except for a single 10 mM CaCl_2_ replicate.

**Table 2:** The values of moments – mean residence time (first moment), variance (second moment), and skewness (third moment) – of the observed effluent breakthrough curves (BTCs) for non-reactive tracer and *Toxoplasma gondii* oocysts in different groundwater chemistry solutions.

Systems	Replicate number	Mean residence time (First moment)	Variance (Second moment)	Standard deviation	Skewness (Third moment)
KBr 10 mM	1	1.312164995	0.003405042	0.058352741	49.58201379
	2	1.434223874	0.002683893	0.0518063	50.18179476
KCl 1 mM	1	1.104383701	0.006237859	0.078980115	18.91386953
	2	1.383736411	0.045893054	0.214226642	55.25992627
NaCl 1 mM	1	1.840036026	0.116739555	0.341671706	59.36036336
	2	1.743875719	0.069404325	0.263447006	48.5264599
NaCl 10 mM	1	1.090428912	0.016863365	0.129859021	36.47529713
	2	1.235291985	0.058543195	0.241957011	27.8973809
CaCl_2_ 1 mM	1	0.856751318	0.046813493	0.216364259	52.10862181
	2	1.044036118	0.183059407	0.427854422	76.60281778
CaCl_2_ 10 mM	1	4.529374782	0.016299222	0.127668406	−11.9040178
	2	1.377010337	0.158701791	0.398373934	54.36172705
MgCl_2_ 1 mM	1	1.047691252	0.058371495	0.241601934	58.11653785
	2	1.324643	0.202644768	0.450160825	80.76930655
MgCl_2_ 10 mM	1	1.298796453	0.052238722	0.228557919	28.69360861
	2	0.851465736	0.270555468	0.520149467	120.9916168
Humic acid 1 mg/L	1	1.044309598	0.017518916	0.132359042	46.7170739
	2	1.140512182	0.039469321	0.198668874	50.69089928
Humic acid 10 mg/L	1	1.158796471	0.038189187	0.195420538	57.63782574
	2	1.126088979	0.035976505	0.189674735	55.66742502
Fulvic acid 1 mg/L	1	0.938337751	0.04387581	0.209465534	45.65594584
	2	1.291501083	0.094242306	0.306989097	70.80947307
Fulvic acid 10 mg/L	1	1.238071474	0.056786527	0.238299239	61.44103786
	2	1.065025971	0.049877652	0.223333051	61.27740164
CaCl_2_ 1 mM + Humic acid 1 mg/L	1	1.374363029	0.056797903	0.238323107	37.05868505
	2	1.041473902	0.101481244	0.318561209	67.09047799
CaCl_2_ 10 mM + Humic acid 1 mg/L	1	1.016728434	0.048144795	0.219419221	38.75817187
	2	3.138627957	0.060692844	0.246359176	2.062255382

### Effects of dissolved organic compounds, alone or in combination with CaCl_2_, on the oocyst transport

Oocyst breakthrough occurred prior to the peak of the KBr tracer curve for both humic acid concentrations (1 and 10 mg/L) (Fig 5A and 5B). Oocysts in 1 mg/L humic acid solution were detected at 0.35 to 0.66 PV and peaked from 1.08 to 1.29 PV, with concentrations ranging from 2.3 x 10^−2^ and 1.2 x 10^−1^ C/C_0_, prior to decreasing between 5.5 x 10^−5^ and 4.6 x 10^−4^ C/C_0_ (Fig 5A and Table 1). The 10 mg/L humic acid solution yielded the same graph shape with higher overall oocyst concentrations in effluents (Fig 5B). Oocysts were first detected at 0.35 PV and peaked from 1.07 to 1.28 PV, with concentrations ranging from 6.1 x 10^−2^ to 6.8 x 10^−2^ C/C_0_ (Table 1). Then, oocyst concentrations decreased to range between 4.0 x 10^−5^ and 5.3 x 10^−4^ C/C_0._ The amount of oocysts compared to the total pulse was 9.295% and 9.249% in 1 mg/L and 10 mg/L humic acid solutions respectively (Table 1). Compared to the KCl control solution, the addition of 1 mg/L and 10 mg/L humic acid resulted in oocyst breakthrough of 1,333% and 1,327% respectively (Table 1).

For both fulvic acid concentrations, oocysts peaked in effluents prior to KBr tracer (Fig 6A and 6B). Oocysts in 1 mg/L fulvic acid solution were first detected at 0.35 PV and peaked at 1.08 PV, with concentrations ranging from 4.0 x 10^−3^ to 4.4 x 10^−3^ C/C_0_, prior to decreasing around 3.7 x 10^−4^ C/C_0_ (Fig 6A and Table 1). The 10 mg/L fulvic acid solution yielded the same graph shape with higher overall concentrations of breakthrough (Fig 6B). Oocysts were first detected at 0.35 PV and peaked between 1.08 to 1.29 PV, with concentrations ranging from 1.3 x 10^−2^ to 1.4 x 10^−2^ C/C_0_ (Table 1). This peak was followed by a decrease in oocyst breakthrough to C/C_0_ values between 2.7 x 10^−4^ and 6.0 x 10^−6^ (Fig 6B). The amount of oocysts compared to the total pulse was 1.074% and 2.173% in 1 mg/L and 10 mg/L respectively (Table 1). Compared to the KCl control solution, the addition of 1 mg/L and 10 mg/L fulvic acid resulted in a breakthrough of 154% and 312% respectively (Table 1). Higher concentration of fulvic acid resulted in a greater transport of oocysts in comparison with the lower fulvic acid concentration. Regarding the first moment of the mean residence time of oocyst BTCs, oocysts in fulvic and humic acid solutions appeared sooner than the tracer and control KCl solution (1.0-1.1 PV for humic acid and 0.9-1.3 PV for fulvic acid vs. 1.1-1.4 PV for KCl) (Table 2). On average, the second moment of their BTCs exhibited a greater variance compared with both the KCl control and KBr tracer BTCs (Table 2). The third moment of skewness of the BTCs for solutions containing organic compounds displayed greater positive values (Table 2).

Then, we evaluated the effects of 1 mg/mL humic acid combined with 1 and 10 mM CaCl_2_ on the oocyst transport. Oocyst breakthrough peaked prior to the KBr tracer curve for all columns except one containing 10 mM CaCl_2_ solution with humic acid. For the 1 mM CaCl_2_ + 1 mg/mL humic acid, oocysts were detected at 0.35 PV and peaked at 1.08 PV with concentrations ranging from 2.6 x 10^-4^ to 4.5 x 10^-4^ C/C_0_ prior to decrease around 2.6 x 10^-4^ C/C_0_ (Fig 7A and Table 1). The 10 mM CaCl_2_ + 1 mg/L humic acid yielded the same graph shape with lower overall oocyst concentrations for one column and did not show a defined peak for the duplicate column (Fig 7B). Oocysts were first detected at 0.35 PV and peaked at 1.08 PV, with a concentration of 1.8 x 10^-4^ C/C_0_. This peak was followed by the trailing off of oocyst breakthrough with C/C_0_ values at 6.0 x 10^-5^. The duplicate sample ranged between 1.3 x 10^-6^ and 4.5 x 10^-5^ oocyst C/C_0_ (Table 1). The amount of oocysts in sampled effluents compared to the total pulse was 0.08% and 0.023% in humic acid solution containing 1 mM and 10 mM CaCl_2_ respectively (Table 1). Compared to the KCl control solution, the addition of 1 mM and 10 mM CaCl_2_ in humic acid solution resulted in oocyst breakthrough of 11.5% and 3.3% respectively (Table 1). Increasing the concentration of CaCl_2_ in the humic acid solution from 1 to 10 mM yielded a decrease in the oocyst transport. When comparing the BTCs of CaCl_2_ in both the presence or absence of humic acid, the mean residence time for the BTCs decreased in the 10 mM CaCl_2_-humic acid system (1.0-3.1 PV) compared to 10 mM CaCl_2_ (1.4-4.5 PV) and increased in the 1 mM CaCl_2_-humic acid system (1.0-1.4 PV) compared to 1 mM CaCl_2_ (0.8-1.0 PV) (Table 2). On average, most of BTCs of the humic acid+CaCl_2_ solutions had a greater variance compared with both the KCl control and KBr tracer BTCs (Table 2). The third moment of skewness of the BTCs for such solutions was characterized by positive lower to higher values compared to values for CaCl_2_ and humic acid alone respectively (Table 2).

## Discussion

We studied the role of electrolytes and natural dissolved organic matter in the transport of *T. gondii* oocysts in saturated engineered sand columns and provided new insights into certain mechanisms that could govern the fate of this important zoonotic parasite in naturally porous media. Oocyst breakthrough curves (BTCs) for all column experiments shared similarities regardless of water chemistry, including early breakthrough of the parasites and a positive third moment defined as the skewness of the BTCs. This early breakthrough when compared to the nonreactive KBr tracer suggests that oocyst mobility, in most instances, is faster [36,37]. Similar column experiments using bacteriophage MS2 in saturated porous media in the presence of organic compounds and various ionic strengths identified a similar phenomenon [38,39], possibly due to the pore inaccessibility of the colloids due to their size. A portion of the void volume was also found to be inaccessible to the colloids, which resulted in earlier breakthrough [40]. This pore inaccessibility results in the transport of colloids through preferential flow pathways with faster travel times as the larger colloids travel through central streamlines, increasing their initial breakthrough as compared to a nonreactive tracer.

The addition of electrolytes (KCl, NaCl, CaCl_2_ and MgCl_2_) reduced oocyst breakthrough in a concentration-dependent manner. Additionally, higher ion valency led to a greater reduction in the oocyst breakthrough, especially the divalent cations Ca^2+^ and Mg^2+^. These results are similar to other saturated flow transport experiments of bacteriophages and microspheres [31,39,41], viruses [42] and oocysts of the protozoan *Cryptosporidium parvum* [11,16,23]. The transport of colloids within saturated porous media is governed by advection and dispersion [16] whereas their retention is governed by deposition and straining [43]. The solution chemistry is known to affect the transport of colloids given the effect of the interaction between the colloids and the grain surfaces within the saturated porous media [11], through van der Waals forces, electrostatic interactions, and static forces acting between the grain surface and the colloids [11,20,44]. The influence of ions upon the electric double layer of the surface interactions between one another causes its compression, therefore inducing the deposition of colloids onto the grain surfaces [44]. In turn, such deposition causes compression of repulsive forces and increases the attraction between surfaces. As the ionic strength increases, repulsion between the grain surfaces and the oocysts decreases, thus enhancing retention of the parasites due to attractive interactions… Moreover, divalent ions create a bridging effect between the surfaces to further the repulsive forces that likely results in retention of the parasites as shown for other colloids [43,45]. In view of these studies, CaCl_2_ and MgCl_2_ caused greater retention of *T. gondii* oocysts in porous media, probably due to the capacity of the divalent cations Ca^2+^ and Mg^2+^ to reduce the thickness of the double layer and promote cation bridging [46–52].

The addition of organic matter (humic acid and fulvic acid) into the solutions increased the oocyst breakthrough. Although increasing fulvic acid concentration from 1 to 10 mg/L enhanced oocyst breakthrough, increasing humic acid concentration had a negligible effect. Natural organic matter including humic and fulvic substances have been known to change the physicochemical characteristics of colloids and their retention because of hydrophobic, electrostatic, and steric interactions as a function of the chemistry of environmental systems [23]. The interactions between oocysts, natural organic matter (NOM), and mineral surfaces such as quartz silica sand are critical determinants of oocyst concentration and transport in effluent systems. Adsorption of humic and fulvic acids onto oocyst surfaces and sand particles alters their surface properties by introducing steric repulsion via polymer-like surface coatings and increasing electrostatic repulsion through changes in surface charge density, which can reduce attachment efficiency and favor breakthrough [53]. The presence of humic substances can suppress pathogen adhesion by modifying surface potential, hydration forces, and the energy barrier to deposition [54,55]. Natural organic matter exhibits significant heterogeneity, with certain fractions enhancing colloid stability and mobility while others promote aggregation or retention, leading to complex transport outcomes [56]. Additionally, solution chemistry—including ionic strength, pH, and electrolyte composition—can modulate these interactions by compressing the electrical double layer and decreasing electrostatic repulsion, thereby enhancing deposition in porous media [43]. Beyond these microscale interaction mechanisms, predicting pathogen transport requires consideration of collector efficiency and physicochemical filtration models, which incorporate hydrodynamic and interaction forces governing attachment [44]. Field and laboratory studies demonstrate that pathogen survival and mobility are further influenced by factors such as temperature, microbial competition, and aquifer heterogeneity, underlining the need for comprehensive evaluation in risk assessments [57]. Moreover, a broad understanding of colloid-associated contaminant transport emphasizes the roles of surface chemistry, flow conditions, and subsurface heterogeneity, highlighting the importance of integrating colloid science in contaminant fate modeling [58].

When observing effects of different ionic and organic solutions, oocysts behaved similarly as the individually tested solutes. One mM and 10 mM CaCl_2_ solutions with 1 mg/L of humic acid displayed results between the values of the individual CaCl_2_ and humic acid experiments. The addition of humic acid to CaCl_2_ solutions increased the oocyst breakthrough when compared to CaCl_2_ alone. Compared with the results with humic acid alone, CaCl_2_ led to a reduction in the oocyst breakthrough. The observed breakthrough concentrations in combined CaCl₂–humic acid systems were more closely aligned with those of the CaCl₂-only experiments, indicating that ionic strength exerted a dominant control over oocyst transport behavior within the tested concentration ranges. This suggests that, despite the presence of organic matter such as humic acid, ionic interactions, particularly those involving divalent cations, play a more significant role in governing oocyst retention and mobility under these conditions. Numerous studies support the dominant role of ionic strength, especially in the presence of divalent cations like Ca² ⁺ , in controlling oocyst and colloid transport in porous media. Higher ionic strength compresses the electrical double layer, reducing electrostatic repulsion and promoting attachment to collector surfaces [44,59]. Divalent cations can also bridge negatively charged functional groups on both oocyst surfaces and mineral grains, further enhancing retention [60]. While humic substances can increase colloid stability by steric and electrostatic stabilization [61], their effectiveness diminishes at elevated ionic strengths due to charge screening and cation bridging [62]. Experimental work has shown that even in the presence of humic acid, increased CaCl₂ concentrations lead to greater deposition of *Cryptosporidium* oocysts and other pathogens, underscoring the overriding influence of ionic interactions in determining transport behavior under such conditions [16,25].

In conclusion, electrolytes enhanced the retention of *T. gondii* oocysts in saturated porous media whereas organic matter increased their transport. These effects align with other colloids displaying breakthrough concentrations governed by DLVO forces, including surface charge and steric hindrances. The introduction of organic matter in farming and agriculture systems could therefore favor the spread of oocysts throughout the environment and hosts. Conversely, increased ionic strength of soil chemistry could promote retention of oocysts, thus leading to the concentration and acquisition by hosts of large amounts of infective oocysts at certain points that could be detached or released at a later time. This new knowledge of transport and retention in soil and groundwater could help public authorities to implement effective measures to prevent the risk of dissemination of these infectious agents.
