# Correction: Detection, transport, and retention of *Toxoplasma gondii* oocysts in saturated sandy porous media: Influence of electrolytes and natural organic matter in flow-through systems

**DOI:** 10.1371/journal.pone.0347130

**Published:** 2026-04-13

**Authors:** Christian P. Pullano, Mahsa Ghorbani, Timothy J. Mutty, Sohib Gouasmia, Coralie L’Ollivier, Jitender P. Dubey, Aurélien Dumètre, Christophe J. G. Darnault

There is an error in the caption within Fig 6. Please see the correct Fig 6 here.

**Fig 6:**
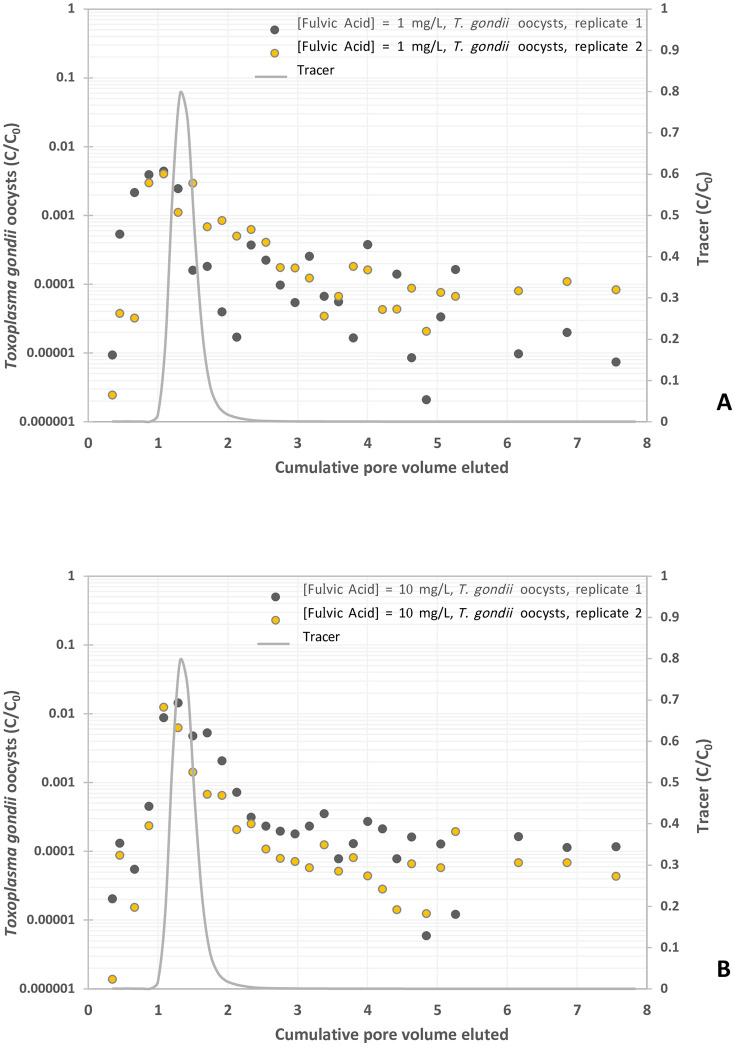
Experimental BTCs of *Toxoplasma gondii* oocysts in suspension of fulvic acid at different concentrations, y-axis represents the normalized effluent concentration C/C0 and x-axis represents dimensionless time expressed as pore volumes, which are equivalent to the volume of fluid injected divided by the total pore volume of the column. (A) Fulvic acid = 1. mg/L, and (B) Fulvic acid = 10 mg/L.
